# Evaluation of emotional excitation during standardized endotracheal intubation in simulated conditions

**DOI:** 10.1186/s13613-018-0460-0

**Published:** 2018-12-04

**Authors:** Nicolas S. Marjanovic, Christelle Teiten, Nicola Pallamin, Erwan L’Her

**Affiliations:** 1 Emergency Department and Prehospital Care, University Hospital of Poitiers, 86000 Poitiers, France; 2 CIC1402 U5 ALIVE – University Hospital of Poitiers, 86000 Poitiers, France; 3 ABS-Lab – Laboratory of Anatomy, Biomechanics and Simulation, Poitiers University, School of Medicine, 86000 Poitiers, France; 4 Emergency Department, University Hospital of Brest - La Cavale Blanche, 29609 Brest Cedex, France; 5 B-com, Institute of Research and Technology, Brest University, 29609 Brest Cedex, France; 6 Department of Intensive Care Medicine, University Hospital of Brest - La Cavale Blanche, Boulevard Tanguy Prigent, 29609 Brest Cedex, France; 7 CeSIM/LaTIM UMR 1101, Western Brittany University – School of Medicine, Rue Camille Desmoulins, 29200 Brest, France

**Keywords:** Patient simulation, Laryngoscopy, Emergency medicine, Emotional excitation, Stress

## Abstract

**Objective:**

To assess how stressful conditions in endotracheal intubation could induct emotional excitation in a population of acute care physicians.

**Materials and methods:**

Two situations were randomly tested: one in standard and easy intubation conditions the other under difficult conditions presumed to induce stress (monitoring alarms, manikin lying on the floor, difficult intubation). Emotional excitation was assessed using several physiological (cardiac patterns, electrodermal activity and eye-tracking) and psycho-cognitive patterns. Auto-evaluations of video recordings and mental workload were performed immediately after simulation.

**Results:**

Significant physiological parameter modifications were observed under the stressful intubation conditions (SDNN 35 ± 15 vs. 42 ± 21; *p* = 0.035—AVNN 514 ± 94 vs. 548 ± 110; *p* < 0.0001). Emotional excitation was demonstrated to lead a higher mental workload (NASA-TLX = 39 ± 18 vs. 63 ± 15; *p* = 0.001), frustration and effort dimensions being its determinant components (*p* < 0.01). Video recording auto-evaluations depicted significant emotional excitation occurrence under the difficult conditions, with few differences according to the operator’s experience.

**Conclusion:**

This study highlights the fact that a stress condition during ETI on a simulation model leads to an important emotional excitation as compared to the neutral condition.

## Background

Endotracheal intubation (ETI) is a standard procedure for the airway management. In emergency medicine (EM), anesthesiology and critical care, it is a challenging procedure associated with severe life-threatening complications, including cardiac arrest. These complications vary according to the operator expertise and depend on several factors, notably in prehospital EM, i.e., unstable medical conditions, environmental requirements, unknown patient medical history [1] and emotional excitation.

Technical skills training has traditionally been modeled on an apprenticeship system, where trainees operated under direct supervision from their seniors. Over recent years, this system has evolved, taking into account security concepts developed in the aerospace sector with simulation and healthcare guidelines from national authorities [2, 3]. Simulation is increasingly used in this setting, and different learning programs exist between juniors and seniors [4].

Emotional excitation that is frequently and improperly called “stress” can be considered as a psychological status that may affect physicians’ performance [5]. While a certain degree of emotional excitation may improve task performance (positive impact), it can also become a threat when the perceived demand outweighs individual resources (negative impact, i.e., “stress”).

We made the hypothesis that stress conditions to IOT would induce a high level of emotional excitation as compared to neutral conditions and that the experience could lead to a lower level of stress.

The main objective of this study was to assess how stressful conditions in ETI could induct emotional excitation in a population of acute care physicians. A second objective was to assess how emotional excitation varied among seniors and juniors.

## Materials and methods

### Study design

This prospective, crossover, randomized study was conducted in situ by the CESIM Santé—LATIM INSERM UMR 1101 research unit at Brest University Hospital (France) between April and May 2016.

A block randomization was used to determine the scenario’s order.

The study was approved by our local ethics committee, and participants were asked to complete a demographic questionnaire and to sign informed consent.

### Participants

The study was conducted with acute care medicine juniors and seniors. Seniors had to be at least one-year experienced, but juniors could have no prior or a few experience in endotracheal intubation on real patients.

### Intubation management

Intubation was performed using an endotracheal tube with 7.0 mm of internal diameters (Mallinckrodt, Dublin, Ireland). The direct laryngoscopy was performed using a Macintosh blade (Size 4). Participants could use a flexible mandrel if needed.

The success of the intubation was secondarily confirmed by an experienced practitioner by a visual control and a lungs’ auscultation.

### Scenarios

Two scenarios of drug overdose were designed and tested before prior to the study, to ensure reproducibility and realistic environmental conditions.

ETI was performed using standard devices of our hospital. All subjects, juniors included, were familiar with these devices. We used an ALS Kelly manikin (Laerdal, Stavanger, Norway) and two standardized recordings of the patient’s monitoring system simulator evolution (LLEAP, Laerdal, Stavanger, Norway):

1. *Neutral condition* The manikin was lying supine on a stretcher that could be adjusted by participants. Vital signs were considered stable [heart rate (HR) = 90 bpm, blood pressure = 120/80 mmHg, oxygen saturation (SpO2) = 98%, normal sinus rhythm]. Endotracheal intubation was to be free of any difficult intubation-associated factors. No evolution of the scenario was to occur during the intubation procedure.
2. *Stress condition* The manikin was lying supine on the floor, in a corner of the room, with reduced available space. Patient’s clinical condition was to deteriorate gradually within 2 min: from tachycardia, to atrial fibrillation and ventricular tachycardia; hypoxemia was present at the beginning of the scenario, despite adequate preoxygenation, and was to deteriorate (SpO2 decrease from 92 to 70%). The tongue of the manikin was blown up to Cormack class IV. Deterioration of physiological parameters was to trigger the monitor’s range alarms after a 15-s period, and the alarm volume was set to be very loud.

Physicians handled the scenarios consecutively, in a randomized and crossover design. Participants were informed that ETI was the main purpose of the study and that global care was not required. Preoxygenation and rapid sequence induction were considered already carried out. ECG and pulse oximetry monitor, and all necessary devices were placed beside the manikin, and if required, operators could ask for a gum elastic bougie.

A limit of two unsuccessful attempts and/or a maximum duration of 5 min were considered to define ETI failure.

### Emotional excitation assessment

Emotional excitation was assessed using various physiological and psycho-cognitive parameters:

1. Heart rate variability (HRV). HRV offers a noninvasive indicator of autonomic nervous system activity [6] and was recorded using a two-lead electrocardiogram attached to the upper chest. The signal was sampled at 1000 Hz using a Biopac^®^ MP36R system and analyzed using AcqKnowledge software (*Biopac Systems Inc, Santa Barbara, CA, USA*). All data were visually checked for quality control. Time-domain and frequency-domain analyses of beat-to-beat RR intervals were performed according to the following parameters [7]:
  Heart rate (HR), the average of RR intervals.
  Maximum heart rate (maxHR).
  Standard deviation of RR intervals (SDNN). SDNN reflects all the cyclic components responsible for variability in the period of recording and therefore represents total variability.
  Average of all RR intervals (AVNN).
  LF/HF ratio, ratio of low (LF) to high (HF) frequency power. HF is the power spectral density of series of the RR intervals between 0.15 and 0.40 Hz due to parasympathetic activity, and LF is the power spectral density for intervals between 0.04 and 0.15 Hz due to sympathetic activity. LF/HF ratio. LF/HF ratio was proposed as an index of sympathetic to parasympathetic balance of heart rate fluctuation [8].
2. Electrodermal activity (EDA). EDA can also be used to examine implicit emotional responses that may occur with or without conscious awareness (i.e., threat, anticipation) [9]. An Embrace wristband (Empatica, Cambridge, MA, USA) was used to monitor EDA via skin conductance level (SCL). SCL is a tonic level of electrical skin conductivity in EDA activity [10] and is presumed to reflect sympathic nervous system activity.
3. Eye-tracking. Eye-tracking is a technique that provides an objective, quantifiable and measurable link between an individual and his immediate environment [11]. SMI eye-tracking glasses (SensoMotoric Instruments GmbH, Teltow, Germany) recorded saccades, blinking and eye fixations using iView ETG 2.2 and BeGaze 3.5.101 software (SensoMotoric Instruments GmbH, Teltow, Germany) to record and export data into video and text formats. SMI glasses were adjusted and calibrated individually, before each simulation. Calibration was carried out by aligning the focus circle as displayed on the output screen of the eye tracking notebook with the actual fixation focus of the participant. Data were subsequently analyzed using a proprietary software. Blinks and fixations were recorded in the central vision. Periods with excessive noise, usually due to movement, were eliminated from analysis. Blinks and fixations were expected higher in a stress condition.

### Psycho-cognitive evaluations

Psycho-cognitive evaluations were performed using three different methods after each scenario:

1. Likert-type scale. Each practitioner evaluated stress level using a Likert-type scale, from 1—none—to 10—very important stress
2. Mental workload. Mental workload estimation was assessed using the National Aeronautics and Space Administration Task Load Index (NASA-TLX) [12, 13]. This questionnaire is regularly used to evaluate different medical and surgical procedures and to assess human–machine interface (HMI).
3. Self-assessment stress evaluation. It was carried out immediately after each scenario, while viewing back-recorded video sequences. The participant was asked to quantify his stress level from 0 to 100 using a haptic 3D wheel navigator (3Dconnexion Space Navigator) and a proprietary software (Autoconfrontation V1.0.4, BCom, Brest, France).

### Statistical analysis

Statistical analysis and data entry were performed using SPSS 23.0 software (IBM Corporation, Armonk, NY, USA). Continuous quantitative data are presented as means ± STD. Non-normally distributed data are presented using median and extreme values, categorical variables by a percentage. Statistical analysis was performed using the Wilcoxon test in between groups and the two scenarios for the same population. P-values equal or below 0.05 were considered significant.

## Results

**Fig. 1:**
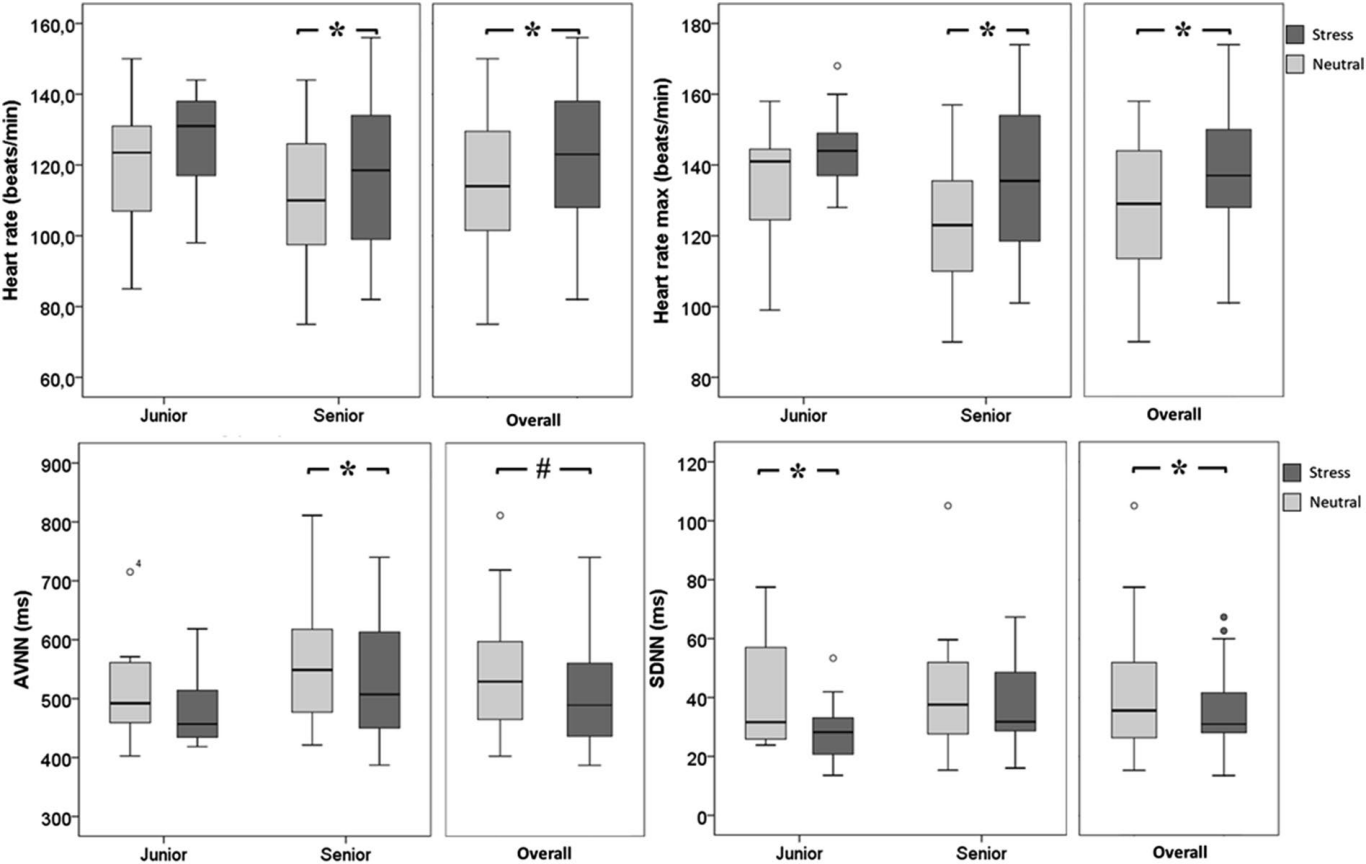
Heart rate and heart rate variability measurement. AVNN is the average of RR interval, and SDNN is the standard deviation of RR. There was a difference in heart rate, max heart rate and AVNN for overall participants and for seniors, between stress and neutral scenarios. For juniors, there was a difference only for SDNN. *p < 0.01 # *p* < 0.0001. HRV is primarily dependent on the parasympathetic autonomic nervous system. Overall, there was a lower HRV between the 2 scenarios with the acceleration of HR, a SDNN and AVNN lower values. Statistical analysis shows significant results in different HRV parameter but not always at the same time

**Fig. 2:**
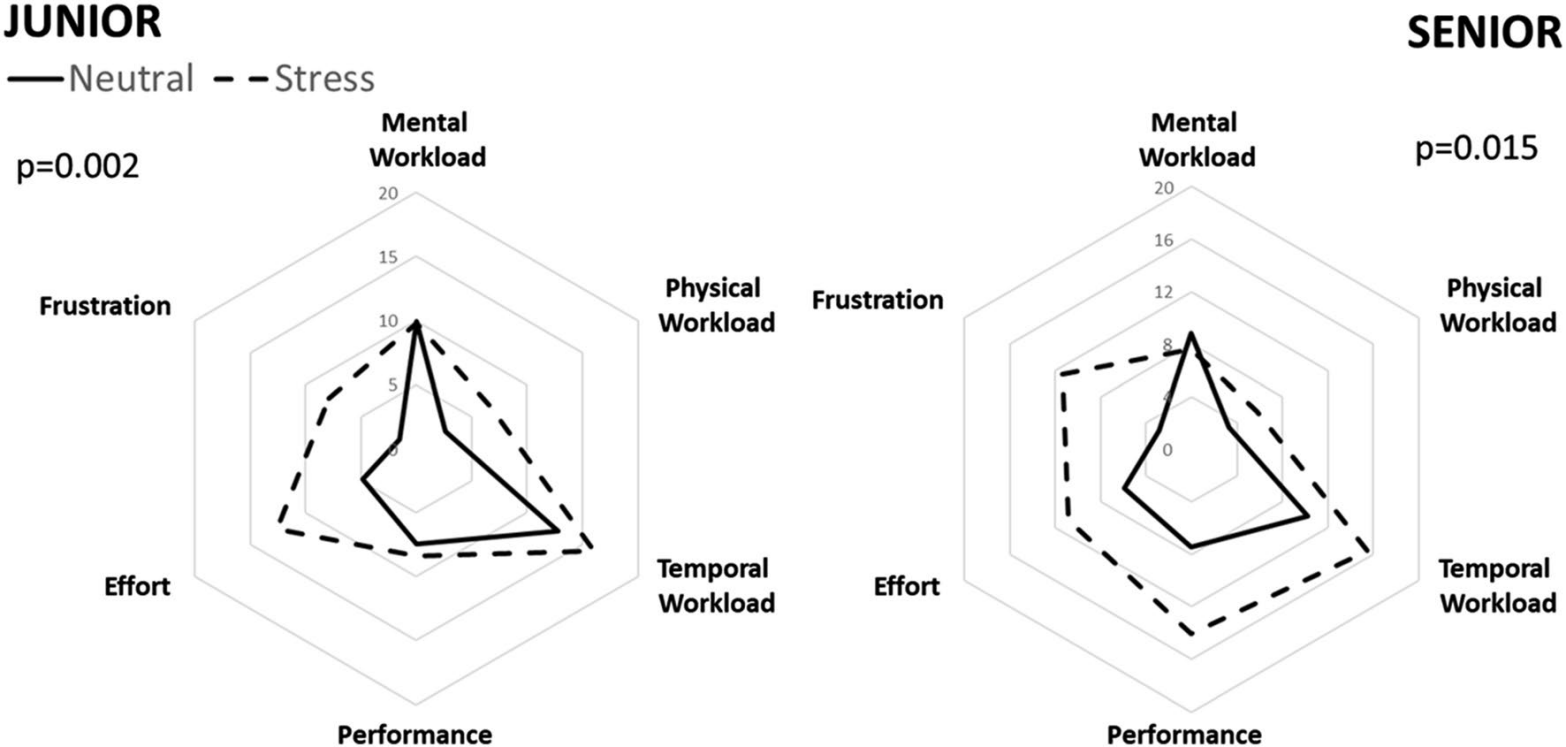
NASA-TLX dimension assessment. Left graph represents juniors’ assessment, and right graph senior’s assessment. Plain line represents measurements during neutral scenario, and dotted line measurements during stress scenario. *p* value compared NASA-TLX between each scenarios and for each group. There was a difference of NASA-TLX between neutral and stress scenario for juniors (38.5 ± 19.7 vs. 65 ± 16.6; *p* = 0.002) and for seniors (39.2 ± 15.7 vs. 61.3 ± 12.9; *p* = 0.015)

Twenty-six acute care physicians were recruited (16 seniors, 10 juniors). Their general characteristics are depicted in Table 1. Physiological patterns are shown in Table 2 and Fig. 1, and psycho-cognitive and subjective assessments are shown in Table 3 and Fig. 2.

**Table 1:** Population characteristics

Age (years)	32.5 ± 6.8
Male *n* (%)	11 (42)
Position	
Seniors *n* (%)	16 (62)
Juniors *n* (%)	10 (38)
Initial formation	
Emergency physician	19 (73)
Anesthesiologist	3 (12)
Intensivist	4 (15)
Intubation experience	
Seniors (years)	11.2 ± 3.7
Juniors (years)	2.4 ± 2.3
Drugs consumption *n* (%)	
Caffeine in the previous 2 h	16 (62)
Anxiolytics	0 (0)
Beta-blockers	0 (0)

Values are provided as mean + STD, or number and percentage

**Table 2:** Physiological measurement of emotional excitation during scenarios

	Overall			Senior			Junior		
	Neutral	Stress	*p*	Neutral	Stress	*p*	Neutral	Stress	*p*
Heart rate variation (HRV)									
HR mean value	114.1 ± 21	120.7 ± 19.9	*0.002*	111.1 ± 21.6	117.2 ± 21.5	*0.007*	119.8 ± 19.8	127 ± 15.8	0.235
HR maximal value	127.1 ± 20.5	140.1 ± 20.6	*0.003*	123.1 ± 21.1	137.8 ± 23.8	*0.005*	134.6 ± 18.3	144.3 ± 13.6	0.362
SDNN (ms)	41.7 ± 21.3	35.1 ± 14.8	*0.035*	41.9 ± 22.2	38.1 ± 15.5	0.307	41.3 ± 21	29.7 ± 12.4	*0.017*
AVNN (ms)	547.9 ± 110.1	514 ± 94.2	<* 0.0001*	563.8 ± 116.8	532.4 ± 103.9	*0.003*	517.9 ± 96.2	481.1 ± 67	0.069
LF/HF ratio	3.5 ± 0.3	3.5 ± 0.2	0.1	3.5 ± 0.3	3.5 ± 0.2	0.256	3.5 ± 0.3	3.6 ± 0.1	0.208
SCL electrodermal activity (ms)	1.8 ± 3.1	1.9 ± 3.1	0.259	2.3 ± 3.8	2.6 ± 3.7	0.721	0.9 ± 0.7	0.7 ± 0.7	0.108
Eye-tracking									
Blink (*n*)	1537 ± 2173	4109 ± 3750	*0.03*	1722 ± 2544	5081 ± 4086	*0.013*	1085 ± 1149	2407 ± 1191	*0.025*
Fixation (*n*)	2451 ± 1752	3806 ± 2249	*0.03*	2023 ± 1423	4002 ± 2381	*0.011*	3339 ± 1980	3537 ± 1951	0.779

Values are provided as mean ± STD or number and percentage

A *p* value ≤ 0.05 was considered statistically significant (in italics)

According to our results, the intubation stress sequence was associated with different HRV parameters, an increase in eye-blinking and fixation episodes in stress condition, as compared to the neutral condition

*HR* heart rate, *HRV* heart rate variability, *SDNN* standard deviation of all NN intervals, *AVNN* average of all NN intervals, *LF/HF ratio* ratio of low (LF) to high (HF) frequency power, *SCL* skin conductance level, *Blink* the overall number of eye links during the measurement periods, *Fixation* the overall number of eye fixations during the measurement periods

**Table 3:** Psycho-cognitive and subjective emotional excitation assessment

	Overall			Senior			Junior		
	Neutral	Stress	*p*	Neutral	Stress	*p*	Neutral	Stress	*p*
Global mental workload	38.8 ± 18	63.5 ± 15.1	<* 0.0001*	38.5 ± 19.7	65 ± 16.6	*0.002*	39.2 ± 15.7	61.3 ± 12.9	*0.015*
Mental workload	9.2 ± 8.3	8.5 ± 9.4	0.922	8.8 ± 9.4	7.6 ± 9.4	0.78	9.9 ± 6.6	9.8 ± 9.8	0.678
Physical workload	3.1 ± 4.2	6.1 ± 5.5	0.023	3.3 ± 4.2	5.8 ± 5.6	0.133	2.7 ± 4.2	6.7 ± 5.7	0.069
Temporal workload	11.2 ± 8.2	15.7 ± 7.5	*0.005*	10.2 ± 8.2	15.5 ± 7.8	*0.036*	12.8 ± 8.3	16 ± 7.4	*0.038*
Performance	7.5 ± 6.7	11.8 ± 8.2	0.089	7.5 ± 7.6	14 ± 8	*0.041*	7.4 ± 5.1	8.3 ± 7.6	0.674
Effort	5.5 ± 3.4	11.5 ± 7	*0.0003*	5.9 ± 3.9	10.8 ± 6.5	*0.003*	4.8 ± 2.4	12.6 ± 8	*0.036*
Frustration	2.4 ± 4.6	10 ± 9.6	*0.003*	2.9 ± 5.5	11.4 ± 9.8	*0.013*	1.5 ± 2.3	7.9 ± 9.4	0.068
Stress measurement									
Likert scale	2.4 ± 1.7	5.3 ± 2.5	*0.00002*	2.3 ± 1.9	5 ± 2.8	*0.009*	2.6 ± 1.6	5.8 ± 2.2	*0.007*
Stress mean	22.3 ± 18.2	45 ± 22.1	*0.000036*	20.2 ± 17.3	44.4 ± 22.3	*0.001*	25.8 ± 20	45.8 ± 22.9	*0.009*
Stress max	38.7 ± 24.6	67.7 ± 26	*0.000033*	35.8 ± 25.8	66.8 ± 26.1	*0.001*	43.2 ± 23	69.3 ± 27.4	*0.009*

In NASA-TLX assessment, each dimension could be analyzed as follows: mental workload: mental and perceptual activity required during task. Physical workload: physical (muscle) activity required. Temporal workload: time pressure felt during task. Performance: feeling of success. It is important to indicate that the performance scale is inversed. The higher the performance dimension, the lower the feeling of performance. Effort: global difficulty (physically and mentally) to accomplish the task. Frustration: level of irritation stress and frustration. The first three dimensions (mental workload, physical and temporal demands) are related to constraints imposed on the subject by the task; the subsequent three other dimensions are related to interactions of the subject with the task. Stress level was assessed according a Likert scale from 1 to 10, and by a video recording auto-evaluation scale from 1—minimum—to 100—maximum

Significant *p* values are highlighted in italics

### Emotional excitation between scenarios

Physiological patterns as HR, maxHR, SDNN and AVNN differed statistically between neutral and stress condition (Table 2, Fig. 1). SCL did not differ between scenarios. The number of blinks and eye fixations was statistically higher in stress condition.

Task load index significantly differed, and subjective assessment of stress felt during scenarios was statistically higher during stress condition.

### Emotional excitation in each group

HR, maxHR and AVNN differed statistically between neutral and stress conditions for seniors (Table 2). We did not observe a difference in HRV patterns between neutral and stress condition for juniors, except with SDNN.

SCL did not differ between scenarios for seniors and juniors, and the number of eye fixations was statistically higher in stress condition for seniors.

In the TLX assessment, temporal workload and effort dimensions were higher during stress condition for juniors, while performance and frustration dimensions likewise differed for seniors as compared to neutral condition.

### Intubation success

In neutral condition, 96% of the participants succeed to perform intubation after the first attempt and 100% after the second attempt. In stress condition, 15.5% succeed after the first attempt, 61.5% after the second attempt, and 23% failed to perform intubation.

## Discussion

This study highlights the fact that a stress condition during ETI on a simulation model leads to an important emotional excitation as compared to the neutral condition. While emotional excitation yet occurred for all participants, unlike studies performed in surgery [14], the data collected in this study suggest that in such conditions, experience has a limited impact on emotional excitation.

Acute care medicine can be considered as a complex system where emotional excitation is one element, interacting with other components such as task performance, such interactions being responsible for human errors [15]. Medical simulation is a powerful tool to assess the different features of emotional excitation and to measure interactions between practitioners and tasks. Our study design depicts a comprehensive approach of emotional excitation, where each physiological and psycho-cognitive data enable to explore specific responses to stress.

HR reflects emotional excitation among practitioners [15]. HR is higher during the first few minutes of the simulation [15–17] and subsequently decreases, possibly due to the practitioner’s getting used to the simulation exercise. It is worthwhile to note that most of the practitioners had high HR, even exceeding 80% of their theoretical maximal value. In our study, HR was significantly higher during stress condition than neutral condition which is consistent with the literature [18]. Heart rate variability (HRV) is a physiological phenomenon, which is a suitable assessment of the autonomous nervous system controlling the heart functions, thus exploring another aspect of HR in emotional excitation.

Considering that cardiovascular activity is directly influenced by the autonomic nervous system, LF/HF ratio has been proposed as an index of sympathetic to parasympathetic balance of heart rate fluctuation. HF is driven by respiration and appears to be an indicator of the parasympathetic activity, while LF is derived from both parasympathetic and sympathetic activity. HRV was also explored through AVNN and SDNN measurements, which are presumed to be less dependent from emotional excitation. Our data therefore suggest a disturbed autonomic nervous system function during simulation [19]. Such a phenomenon has been associated with mental stress [20] and suggested to indicate inability to respond to physiological variability [21]. Senior HRV tends to be higher than junior ones, which may raise the hypothesis that experience is responsible for a more tranquil behavior, as also observed in other studies [18, 19, 21]. During HRV frequency analysis, low HR values were responsible for a higher LF/HF ratio, the persistence of this indicator within scenarios indicating sympathetic system activation, even in a neutral situation [22]. Short-lasting exposure to psychosocial stressors has been described to indicate parasympathetic withdrawal and increased sympathetic activity [23].

Emotional excitation induces an increase in the sweat gland activity and therefore a modification of the electrical properties of the skin (EDA), which can be assessed by SCL [24]. However, SCL values did not differ in between scenarios within our study. Wachtel et al. reported that in the event of stress, some participants were slower to respond to peripheral stimuli [25]; it could therefore be considered that the delay in between the scenarios was too short in our study. While 10–25% of our participants were estimated as non-responders in terms of EDA [26], some participants may have cardiovascular abnormalities, which may lead to EDA rhythmic artefacts [9]. Finally, it is not possible to obtain high-quality EDA measurements from some individuals, especially in simulation conditions with movement artefacts [9].

Another way to assess stress is eye-tracking techniques, which provides an objective, quantifiable and measurable link between an individual and his immediate environment [11]. Because links between the focus of vision and the focus of cognition have been established [27], eye-tracking may provide useful information for studying situational awareness [28]. As expected, eye fixation and number of blinks were higher in stress conditions, suggesting an enhanced encoding of the environment [29] in order to improve performance. However, juniors had more eye fixation in neutral conditions than experienced practitioners, and they did not experience more fixation during stress condition. As compared to juniors, seniors had more eye fixations in stress conditions. Herten et al. showed that emotional excitation leads to a memory enhancement and to promote attentional narrowing toward relevant stimuli at the expense of irrelevant data, associated with more eye fixation [29]. Schulz et al. have demonstrated that juniors were spending more time looking at the patients’ monitoring devices than experts [30]. They suggested than experienced practitioners might be able to maintain sufficient situation awareness without spending more time in monitoring tasks and could actively direct their attention toward manual and relevant tasks [30]. Our results suggested stress condition was not associated with a memory enhancement for juniors, probably due to their inexperience. In normal condition, we suspect than eye fixation was linked with an inappropriate attentional narrowing on irrelevant data. By contrast, because of emotional excitation, seniors were able to better optimize their memory to improve their performance than juniors, resulting with more eye fixation in stress condition. Attentional narrowing in a stressful situation may be related to a greater fixation [31, 32], thus being a predictor of memory performance [33]. While stress seems to prepare the organism for accurate attentional processing of relevant stimuli, eye-tracking technology has the potential to transform the way clinical simulation is evaluated, thereby helping to improve patient safety [34]. Its standardized use may lead to the development of training protocols to improve students’ education.

According to our participants, the stress condition generated more emotional excitation and more mental workload than neutral condition. Task load index, assessed by NASA-TLX, is a psycho-cognitive scale measuring difficulty to perform a task [13]. In the NASA-TLX measurement, TLX is influenced by two types of dimension: (1) dimensions depending on human–machine interface (mental, physical and temporal workload) and (2) dimensions depending on the task and on the environment of the task (effort, performance and frustration). Within our study, the importance of each dimension differed in between groups. For seniors, the difference mainly concerned the performance and frustration, while for juniors there was a trend toward physical workload increase. These results could be explained by the fact that experience diverts the focus of participants when they are subjected to emotional excitation. Juniors are concerned by procedure itself, while seniors are concerned by success, which can generate frustration.

Our study suffers from limitations. First, while this was an exploratory study, no power calculation was performed to assess sample size and a post hoc analysis may consider that the observed power was insufficient to allow conclusion on the secondary objectives of the study. Moreover, while practitioners were recruited at their workplace, individual stress factors independent from our study could have been exerted on participants. Second, heart rate variations might be influenced by physiological demand that could differ between scenarios. However, this does not seem to be a major problem for results analysis while: (1) HRV variations are not strictly correlated with heart rate values; (2) physiological demand during the difficult intubation scenario was calibrated to be as lower as possible for the physicians. Furthermore, other recorded parameters (that cannot be correlated with physiological demand) were consistent with an increased emotional excitation in between the two different scenarios: (1) eye-tracking assessment of blinking and eye fixation; (2) psycho-cognitive scoring using Likert scales and NASA-TLX. Third, the motion sensor clusters distributed among the practitioners were a key factor limiting their freedom of movement; the participant was alone to perform the task, while in clinical routine he will be provided some help and use several other difficult intubation equipment. Finally, the choice and interpretation of our sensors results could also be discussed. Concerning eye-tracking, Lotfus et al. concluded that attention and eye fixation could be dissociated [34]. Beside these limitations, our study has some strengths. To the best of our knowledge, this study provides various aspects of emotional excitation, including physiological measurement (HR, eye-tracking and SCL), psycho-cognitive evaluation and subjective stress assessment. Thus, our model is the first to allow a global and comprehensive model assessing emotional excitation due to stressful conditions in acute care medicine procedure.

In conclusion, an isolated analysis of intubation during a simulation is an important source of stress for seniors and juniors when it is presented as a difficult intubation according to prehospital criteria. According to the Yerkes–Dodson law, subjects may need some part of stress to improve performance, while a too high level of stress may decrease performance [35]. Our study depicts significant physiological and psycho-behavioral changes between ETI conditions. Medical simulation taking into account emotional excitation sensors may enable an enhancement of practitioners’ performance while improving teaching.

## Abbreviations

AVNN: average of RR intervals
EDA: electrodermal activity
EM: emergency medicine
ETI: endotracheal intubation
HMI: human machine interface
HRV: heart rate variability
maxHR: maximum heart rate
MWL: mental workload
SCL: skin conductance level
SDNN: standard deviation of HRV
TLX: task load index
